# Case of Traumatic Tetanus

**Published:** 1878-12

**Authors:** Gustav Keitz

**Affiliations:** 666 North Rampart St., New Orleans, La.


*A Case of Traumatic Tetanus.*

Manuel Arrsend, a creole boy, aged 11 years 9 months, was attacked by tetanus on the 16th of September, 1878. I was called on the 19th, 2 p. m.

The boy had picked up a splinter with his left foot on the 8th of September. The splinter had been extracted by his grandmother, an old lady of about 65 years of age, the wound washed, bandaged up with rags, and left to take care of itself. Healed well.

When I arrived at the house, I found traumatic tetanus well developed. Urine very scanty and highly colored; about 3½ oz. were let in 10 hours; skin hot and dry, pulse 136, temp. 39.5, respiration 30, great rigidity of all muscles.

I opened the bowels with the following injection, as they had not moved once in three days:

℞ Extr. sennæ liquidi, salis amari, āā, 25.0
Aq. menthæ pip. 200.0, M. D. S. enema.
Use one-half at once, the rest in 2 hours.

The following mixture was also prescribed:

| | |
|---|---|
| ℞ Potass. bromid. | 15 |
| Hydrati chloralis | 8 |
| Aquæ menthæ pip | 120 |

M. D. S. Tablespoonful every 2 hours, or when required.

The enema was administered at once, and operated three times well. The mixture I administered myself. Putting one corner of the mouth gently aside by means of the handle of a teaspoon, I succeeded in making the patient swallow two tablespoonfuls at once, dropped slowly between teeth and lips. Spasms allayed almost instantly. A flax seed poultice (hot) was ordered to the cicatrix of the wound. On my return at 7 p. m., I found the patient quiet. Pulse 136, temp. 39.5, respir. 30.

Sept. 20th, 7 a. m. The patient had passed a quiet night; spasms were promptly controlled by chloral. Passed seven ounces of urine; urine red, otherwise natural. Pulse 128, temp. 39.2, respiration 30.

As a drink I ordered carbonic acid (Seltzer) water; also, the following liniment:

| | | |
|---|---|---|
| ℞ | Tinct. radicis aconiti, | |
| | Chloroformyli, āā........................... | 15 |
| | Spir. camphoræ............................. | 100 |

M. D. S. Sponge body and limbs every 3 hours.

Five p. m. Patient complained of burning whenever the liniment was applied. This was, therefore discontinued, and the following salve used in its place:

| | | |
|---|---|---|
| ℞ | Ungti mercurialis cinerei (fortioris)....... | 8 |
| | Extracti belladonnæ......................... | 4 |
| | Ungti camphoræ............................. | 30 |

M. ft. ungt. S. Apply 4 grams to the abdomen every 3 hours.

Sept. 21st, 7 a. m. Patient had passed a good night; had urinated twice naturally. Temp. 38.8, pulse 118, respiration 26. I ordered the following mixture:

| | | | |
|---|---|---|---|
| ℞ | Extracti physostigmatis venenosi .......... | 0 | 07 |
| | Pulv. gi. mimosæ, qs. | | |
| | Aquæ menthæ piperitæ...................... | 100 | |

S. Teaspoonful every two hours.

The ointment was discontinued, and the liniment used again. Muscles still rigid.

Five p. m. The patient has been in a continual slumber since this morning. He was easier this evening than at any time previous. Muscles of the face less rigid. I ordered Liebig's beeftea. The liniment had to be discontinued again and the ointment used.

Sept. 22d, 7 a. m. Patient had rested well during the night; urinated twice naturally. Pulse 112, temp. 39.2, respiration 26. No pain; deglutition quite easy. 5 p. m. No change since morning; everything continued.

Sept. 23, 7 a. m. Patient had rested well, and could separate his teeth sufficiently to make the tip of his tongue protrude. Muscles in general less rigid; no pain. The bowels were opened by injection, as before. Profuse epistaxis had set in at 5 this morning, which lasted about half an hour. Temp. 38.6, pulse 106. Previous treatment continued. 5 p. m. No change since this morning. Patient urinated naturally at 4 p. m., and had two operations of the bowels, which were natural. Beef tea and mineral water continued; chloral mixture decreased to half doses.

Sept. 24, 7 a. m. Profuse epistaxis at 1 a. m.; the blood was very dark. Slight twitching of the muscles was noticed and promptly allayed by a full dose of chloral. The patient could open his mouth fully 3 centimetres, could flex and extend his legs quite easily, and carry the feeding cup to his mouth without help. He urinated several times naturally. Pulse 106, temp. 38.6. 2 p. m. Patient had slept three hours since this morning. I visited him once more at 7 p. m., and finding him getting along well, ordered former treatment to be continued.

Sept. 25th, 7 a. m. Profuse epistaxis at 4:30 a. m. Patient could protrude his tongue one-half inch, and separate his teeth fully two lines. He had urinated naturally during the night, and was able to flex and extend arms and legs freely, and to change his position in bed without help. Temp. 38.6, pulse 106. I called again at 12 m. and 7 p m. No change.

Sept. 26th, 7 a. m. Patient had passed a nice night; passed fæces and urine naturally. Temp. 38.4, pulse 102. He could separate his teeth 7 centimetres. I called at 2 and 7 p. m., and ordered former treatment to be continued.

Sept. 27th, 7 a. m. Found patient sitting up in bed. Had urinated three times and defecated once naturally during the night. Temp. 38.4, pulse 102. He could eat biscuits and milk; muscles of face greatly relaxed. Abdomen still hard, but not tender.

Sept. 28th, 7 a. m. Patient still doing well. Could separate his teeth 7½ lines, and masticate small pieces of cracker. Temp. 37.8, pulse 96.

From this on the patient continued doing well, and could walk across the room with very little assistance on the fifteenth day of medical treatment, viz., Thursday, Oct. 3d. I put him on lactate of iron and cinchonidia, beef tea and light but strengthening diet, and allowed moderate exercise. The hardness of the abdomen soon yielded to an ointment composed of 0.25 grams extract of physostigma venenosum, and 30 grams camphor salve, and Manuel Arrsend will attend his school as usual, as soon as it opens.

GUSTAV KEITZ, M. D.
666 NORTH RAMPART ST.,
NEW ORLEANS, LA., Nov. 4th, 1878.

